# Supplementary material for: Spontaneous ssDNA stretching on graphene and hexagonal boron nitride in plane heterostructures
Source: Nat Commun. 2019 Oct 10;10:4610. doi: 10.1038/s41467-019-12584-w (PMC6787186; doi:10.1038/s41467-019-12584-w)
Supplement: Supplementary file 1 — Supporting Information [file 41467_2019_12584_MOESM1_ESM.pdf]

Supplementary Information for: Spontaneous ssDNA Stretching on Graphene and Hexagonal Boron Nitride In Plane Heterostructures

Binquan Luan* and Ruhong Zhou

Computational Biological Center, IBM Thomas J. Watson Research, Yorktown Heights, NY 10598, USA.

E-mail: bluan@us.ibm.com

Supplementary Notes

The entire simulation system (for Sim-1 and Sim-2) is shown in Supplementary Figure 1, where water (not shown in Figure 1c) is shown transparently in the MSMS representation. The height of the simulation system is about 70 Å that is much larger than the Debye screening length of 10 Å in the 0.1 M KCl electrolyte. During simulations in the NPT ensemble, pressure control was only applied in the direction perpendicular to the plane of the 2D heterostructure. Therefore, the cross section area of the simulation system (or the area of the 2D heterostructure) remained constant, while the box can slightly fluctuate in the third direction (perpendicular to the heterostructure surface) to maintain the constant pressure (1 bar).

The entire 3D simulation systems including water for ssDNA on graphene-only surface (Sim-3) and for ssDNA on h-BN-only surface (Sim-4) are illustrated in Supplementary Fig-

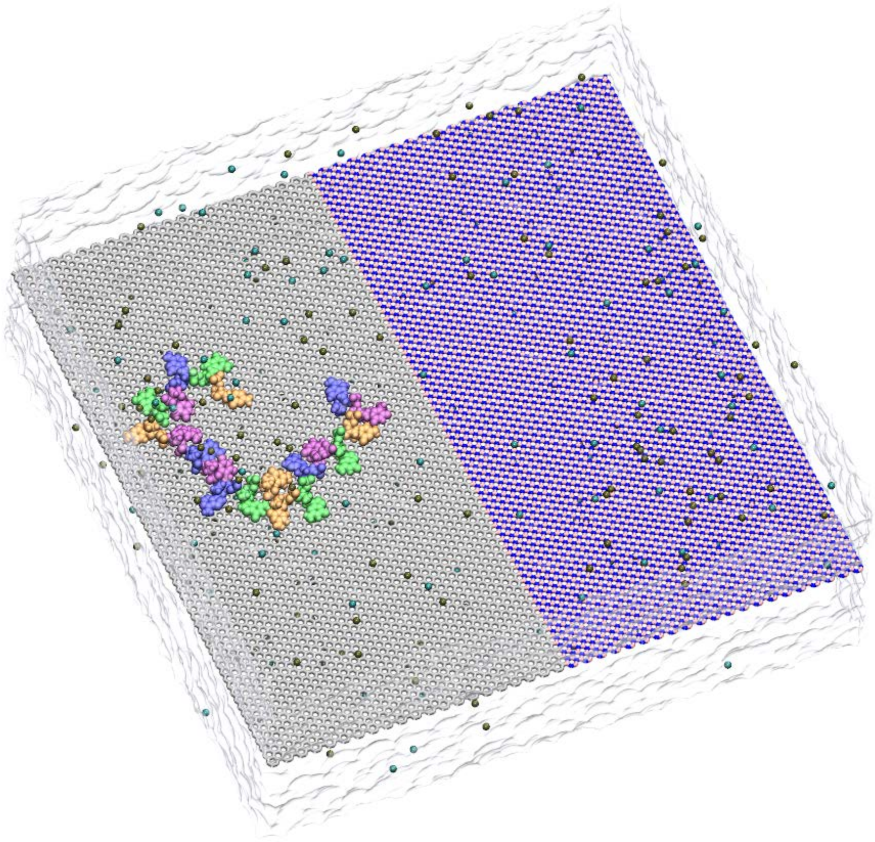

Figure 1: Three dimensional view of the simulation system for ssDNA on the h-BN/graphene heterostructure. Besides the same description as listed in Figure 1 in the main text, here water is shown transparently.

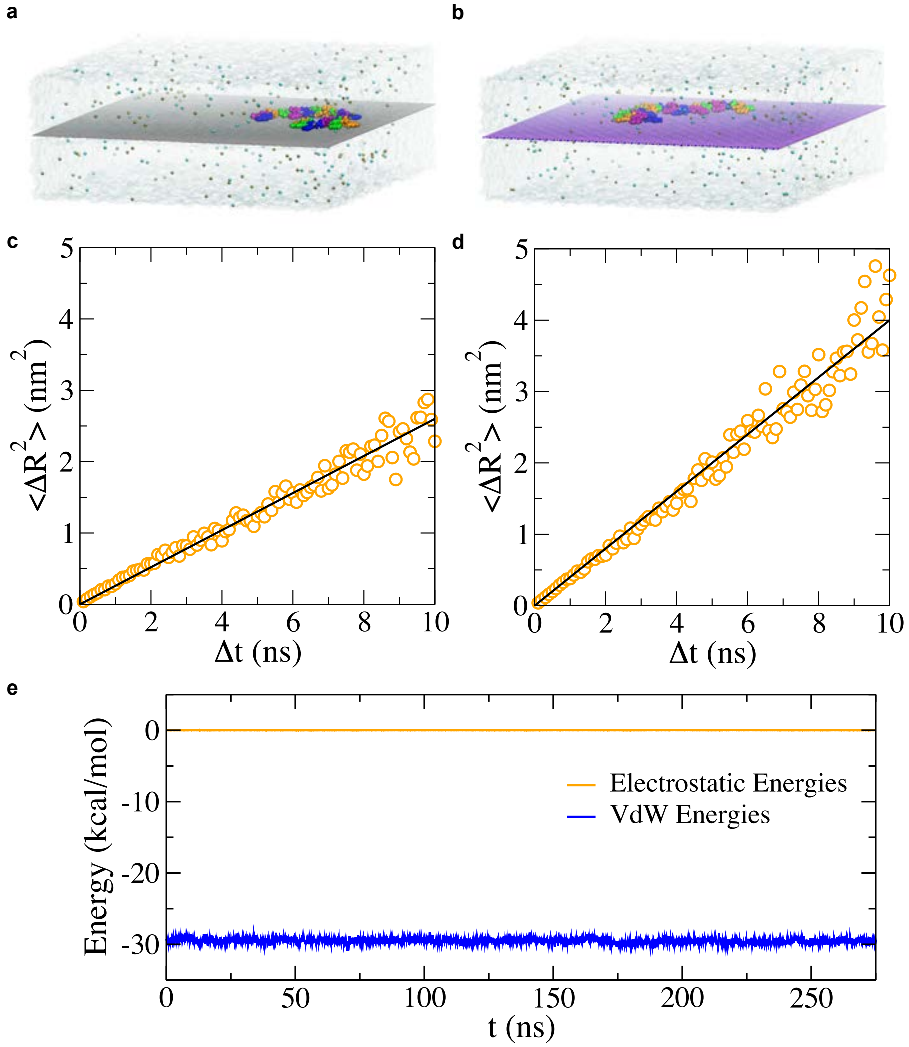

Figure 2: ssDNA diffusion on graphene-only and h-BN-only surfaces. a) Three dimensional view of the simulation system for ssDNA on the graphene. b) Three dimensional view of the simulation system for ssDNA on the h-BN. In a) and b), water not shown in Figures. 1d and 1e in the main text are shown transparently. c) Mean square displacement of ssDNA on the graphene surface vs. time intervals. d) Mean square displacement of ssDNA on the h-BN surface vs. time intervals. e) Electrostatic interaction energies (orange) vs. van der Waals interaction energies (blue) between ssDNA and the h-BN nanosheet.

ures. 2a and 2b. From about 275 ns simulation of each system, we calculated the 2D diffusion coefficient of ssDNA, defined as $D = < \Delta R^2 > / 4\Delta t$ (where $< \Delta R^2 >$ is the mean square displacement and Δt is the time interval).

For ssDNA on the graphene surface $D =$ 26 Å^2/ns, while for ssDNA on the h-BN surface D=40 Å^2/ns. The larger diffusion constant for ssDNA on the h-BN surface could result from the heterogeneous nature of the h-BN surface that makes it even more difficult to lock the ssDNA surface and the h-BN surface into registry (i.e. lower friction).[?]

For carbon atoms in the graphene nanosheet, their charges are set to be zero and thus there is no electrostatic interaction between ssDNA and the graphene nanosheet. In addition, even though charges of boron and nitrogen atoms are +0.4 e and -0.4 e, Supplementary Figure 2e shows that van der Waals interaction energies (-29.3 kcal/mol per nucleotide) between ssDNA and the h-BN nanosheet is significantly stronger than the electrostatic ones (-0.0016 kcal/mol per nucleotide, calculated with the relative dielectric constant 1.0). Therefore, electrostatic interaction energies are negligibly small and can be safely ignored.

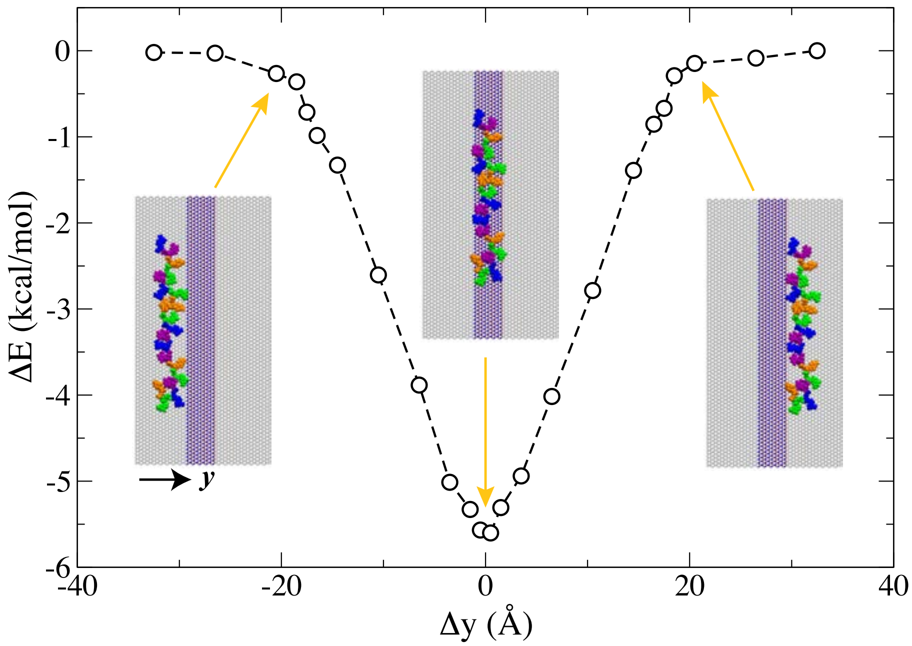

Figure 3: Interaction energy between ssDNA and the 2D heterostructure when a stretched ssDNA moves in the y direction across the h-BN stripe.

To highlight the fundamental mechanism for the observed spontaneous stretching of ssDNA on the h-BN stripe sandwiched by two graphene domains, we arbitrarily chose a stretched ssDNA conformation from Sim-5 and moved it rigidly and horizontally along the 2D heterostructure surface. With the reference energy for ssDNA on the graphene surface (far away from the h-BN stripe), the energy change per nucleotide ΔE decreases gradually when ssDNA approaches the h-BN stripe and later increases again once it leaves the h-BN stripe, forming a potential well for the ssDNA on the graphene/h-BN/graphene surface. Consequently, it is the narrow width of this potential well that leads to the observed stretching of ssDNA.

We considered two cases: (1) the width of a graphene domain $W >>$ the width of a h-BN stripe w and (2) W is a bit larger than w (or comparable).

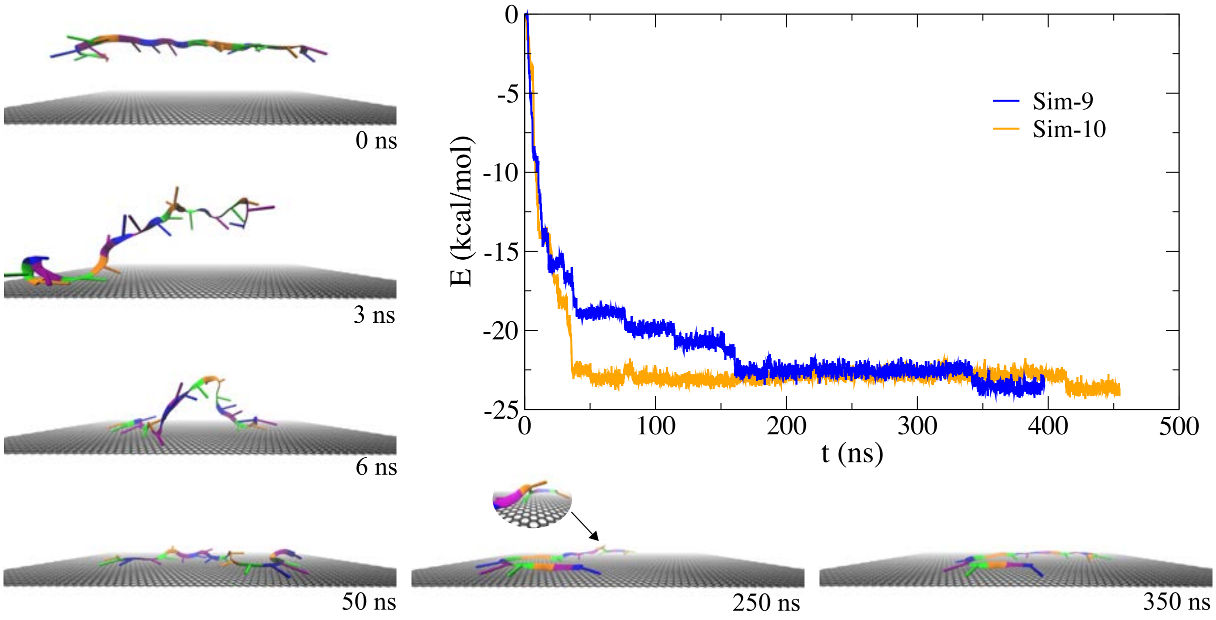

Figure 4: Adsorption of ssDNA from water onto the graphene surface from two independent MD simulations (Sim-9 and Sim-10). Water and ions are not shown for the clarity purpose. Six snapshots were extracted from Sim-9.

For the first case, the ssDNA will be very likely adsorbed on the graphene surface, which is followed by the diffusion toward the h-BN stripe (as shown in the main text).

Therefore, we simulated the adsorption process of ssDNA from water onto the graphene surface. As shown in Supplementary Figure 4, the ssDNA molecule was initially placed about 20 Å above the graphene surface (t=0 ns). Quickly after 3 ns, one end of ssDNA was adsorbed onto the graphene surface while the other end remained in water. After another 3 ns, both ends were present on the graphene surface. Due to the strong π-π stacking, once bases of ssDNA were adsorbed onto the graphene surface, they cannot be released from the graphene surface. After about 50 ns, the entire ssDNA backbone lied down completely. At this phase, the intra-strand base stacking in ssDNA can occur, therefore some ssDNA bases were not adsorbed on the graphene surface (e.g. the snapshot at t=250 ns). Eventually (t=350 ns), all ssDNA bases formed π-π stacking with graphene. Accompanied with more and more adsorbed ssDNA bases, the ssDNA-graphene interaction increased about 23 kcal/mol per nucleotide (more favorable, the blue line in Supplementary Figure 4, Sim-9). In an independent simulation (Sim-10, orange line in Supplementary Figure 4) we observed the similar ssDNA adsorption process, in which the ssDNA-graphene interaction energy saturated at 23 kcal/mol per nucleotide at the end as well.

When the width of a graphene domain is a bit larger than the width of a h-BN stripe, it is likely that the entire (or at least part of) ssDNA molecule can be adsorbed from water to the h-BN stripe directly. To investigate such an adsorption process, we carried out simulations (Sim-11 and Sim-12) with the ssDNA molecule initially placed 20 Å above the graphene/h-BN/graphene heterostructure.

Indeed, we observed the initial adsorption of the ssDNA molecule on or near the h-BN stripe. When T=300 K, we found that after the adsorption, the ssDNA molecule can be partially folded, because of intra-strand base stacking. While such folded structure may only survive for a short period of time on graphene-only surface, the folded structure on the h-BN nanostripe was retained for the entire duration of the simulation (blue line in Supplementary Figure 5, Sim-11). When being physically confined on the h-BN stripe, thermal fluctuation of ssDNA is reduced and the folded structure of ssDNA (see the snapshot of the simulation

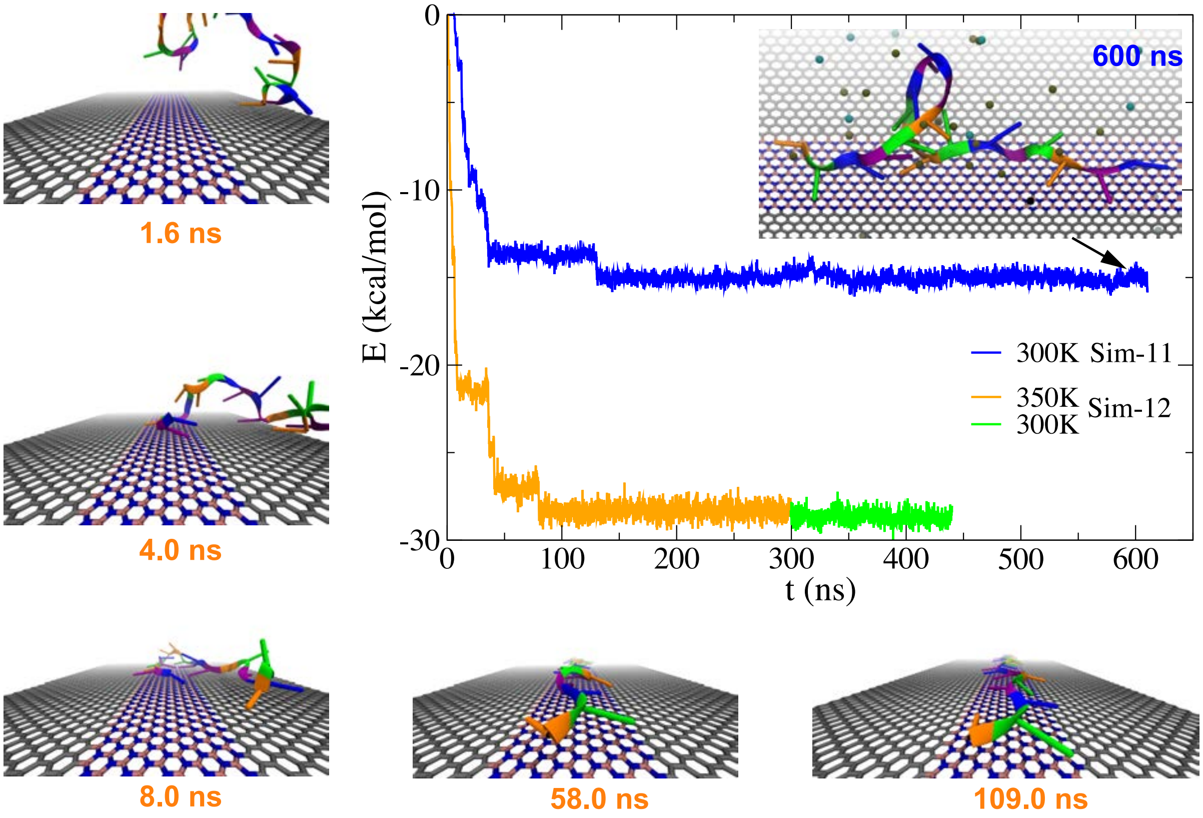

Figure 5: Adsorption of ssDNA from water onto the graphene/h-BN/graphene heterostructure surface from two independent MD simulations (Sim-11 and Sim-12). Water and ions are not shown for the clarity purpose. Five snapshots were extracted from Sim-12 (orange line).

system at t=600 ns, in Supplementary Figure 5 inset) becomes more stable. The presence of folded ssDNA structure prevents ssDNA from being stretched on the h-BN stripe.

To circumvent this difficulty, we raised the temperature to 350 K in simulation (Sim-12, see orange line in Supplementary Figure 5). During the adsorption process, one end of ssDNA landed on the graphene domain at 1.6 ns, followed by the adsorption of the other end of ssDNA on the h-BN stripe at 4.0 ns. Around 8 ns, majority of DNA bases were on the heterostructure surface, with an ssDNA fragment on the graphene surface. Quickly, the entire ssDNA was on the h-BN stripe at 58 ns (similar to phenomenon in Sim-5, Sim-6, Sim-7 and Sim-8, described in the main text). After the removal of an intra-strand base stacking in ssDNA (at 109 ns) through thermal fluctuation, all ssDNA base formed the π-π stacking with the h-BN stripe. The ssDNA molecule remained being stretched in the rest of the simulation. At t=300 ns, we reduced the temperature back to 300 K and simulated the system for additional 140 ns. The interaction between ssDNA and h-BN become slightly stronger resulted from the reduced thermal fluctuation (the green line in Supplementary Figure 5).

Overall, we conclude that ssDNA in water can be adsorbed to the heterostructure surface and further be stretched on the h-BN stripe.

Supplementary Movies

Supplementary Movie 1: showing the simulation trajectory of Sim-5.
Supplementary Movie 2: showing the transport of stretched ssDNA on the heterostructure surface driven by a biasing voltage of 0.2 V.

Supplementary References

(1) Luan, B.; Robbins, M. O. The breakdown of continuum models for mechanical contacts. *Nature* **2005,** ***435*****, 929–932.**
